# *Mycobacterium Ulcerans* Ulcer: Current Trends in Antimicrobial Management and Reconstructive Surgical Strategies

**DOI:** 10.3390/life15071096

**Published:** 2025-07-13

**Authors:** Bryan Lim, Omar Shadid, Jennifer Novo, Yi Mon, Ishith Seth, Gianluca Marcaccini, Roberto Cuomo, Daniel P. O’Brien, Warren M. Rozen

**Affiliations:** 1 Department of Plastic Surgery, Peninsula Health, Melbourne, VIC 3199, Australia; omarshadid4@gmail.com (O.S.); jenny.novo@gmail.com (J.N.); yimon1230@gmail.com (Y.M.); ishithseth1@gmail.com (I.S.); warrenrozen@hotmail.com (W.M.R.); 2 Department of Plastic, Hand & Faciomaxillary Surgery, Alfred Health, Melbourne, VIC 3004, Australia; 3 Plastic and Reconstructive Surgery, Department of Medicine, Surgery and Neuroscience, University of Siena, 53100 Siena, Italy; gianlu32@gmail.com (G.M.); roberto.cuomo2@unisi.it (R.C.); 4 Department of Infectious Diseases, Barwon Health, Geelong, VIC 3022, Australia

**Keywords:** Buruli ulcers, *Mycobacterium ulcerans*, antimicrobials, global health, reconstructive surgery

## Abstract

**Background**: *Mycobacterium ulcerans* causes Buruli ulcer (BU), a necrotizing skin disease endemic in over 30 countries. Its toxin, mycolactone, drives tissue destruction, and the infection is transmitted via environmental reservoirs or vectors. Disease patterns vary globally, and an improved understanding of their pathogenesis may enhance current antimicrobial and surgical treatments. **Methods**: A comprehensive literature search from 1901 to 2025 was conducted across major databases to explore antimicrobial and reconstructive surgical strategies for *Mycobacterium ulcerans*. Search terms included BU, key antibiotics, and surgical interventions. Relevant English-language studies on treatment outcomes were reviewed to summarize evolving management trends and emerging therapeutic approaches. **Results and Discussion**: This review highlights the importance of early diagnosis and timely antimicrobial therapy in preventing disease progression and limb loss. It reviews WHO-recommended antibiotic regimens and discusses the theoretical risk of drug resistance, although clinical resistance remains rare and unreported in Australia. Surgical interventions in select cases are crucial, with timing being a significant factor in functional outcomes. The review also covers pediatric-specific challenges, including growth preservation and psychosocial support for young patients. Reconstructive options focus on limb salvage and staged reconstructions, with multidisciplinary care essential for optimal outcomes. The paper advocates for RCTs to refine treatment protocols, surgical guidelines, and explore emerging antibiotic therapies such as telacebec. **Conclusions**: BU remains a global health challenge, requiring early diagnosis, timely antimicrobial therapy, and surgery in selected cases. Future research will refine treatment and reduce long-term impacts.

## 1. Introduction

*Mycobacterium ulcerans*, the environmental bacterium causing Buruli ulcers (BU), produces necrotizing skin infections and is the third most common mycobacterial disease after tuberculosis and leprosy, mainly affecting immunocompetent individuals [1,2,3,4,5]. First described in Australia in 1948 [6,7], BU now occurs in over 33 countries, with Côte d’Ivoire, Ghana, and Benin accounting for ~73% of global cases, though recent declines may reflect under-reporting [8]. BU is a chronic necrotizing skin disease that mainly affects immunocompetent individuals. These infections are characterized by progressive, painless necrosis of subcutaneous tissue, often resulting in extensive ulceration with undermined edges and minimal systemic symptoms. In Africa, 48% of cases affect children under 15 [8], whereas in Australia, the rising incidence in regions such as Victoria’s Bellarine and Mornington Peninsulas primarily affects immunocompetent older adults in high socio-economic areas [3,4,5]. Despite these demographic differences, BU does not typically exhibit a predilection for immunocompromised individuals. These patterns highlight evolving public health concerns [4,9,10,11].

The transmission of *Mycobacterium ulcerans* remains poorly understood but is thought to involve environmental reservoirs such as contaminated water, soil, or vegetation [10,12,13,14]. In Australia, native possums are considered significant reservoirs, with geographic overlap between BU hotspots and areas where possum feces test positive for the bacterium [10,14]. Mosquitoes may act as potential vectors in this zoonotic cycle. Surveillance of possum excreta has emerged as a promising tool for predicting human cases and guiding public health interventions [5,15].

*Mycobacterium ulcerans* pathogenesis is primarily driven by mycolactone, a polyketide toxin that causes tissue necrosis and immune suppression, enabling the bacterium to persist extracellularly in subcutaneous tissue [16,17,18,19]. These infections are characterized by progressive, painless necrosis of subcutaneous tissue, often resulting in extensive ulceration with undermined edges and minimal systemic symptoms. Advances in inducible expression systems have deepened understanding of mycolactone’s effects on host pathways such as mTOR signaling, autophagy, and apoptosis [20,21], offering new insights into disease progression. These molecular findings are essential for informing antimicrobial therapy and reconstructive surgical approaches [20,22]. Current treatment strategies often fail to incorporate recent advances in knowledge of BU pathogenesis, and surgical practices vary across healthcare settings. This review synthesizes emerging evidence on BU’s molecular mechanisms, antimicrobial management, and surgical approaches to guide future research and improve patient outcomes.

## 2. Materials and Methods

A comprehensive literature search from January 1948 to May 2025 was conducted to examine the evolving trends in antimicrobial therapy and reconstructive surgical approaches for *Mycobacterium ulcerans* and BU. PubMed, Scopus, Cochrane Central, and Web of Science databases were queried using a strategic combination of keywords and medical subject headings (MeSH). Primary search terms included “*Mycobacterium ulcerans*”, “Buruli ulcer”, “antimicrobial treatment”, and “reconstructive surgery”. These terms were further refined using Boolean operators and intersected with secondary terms, including “rifampicin”, “clarithromycin”, “surgical debridement”, “flap reconstruction”, “skin grafting”, “antibiotic resistance”, and “treatment outcomes”. The inclusion criteria comprised systematic reviews, meta-analyses, clinical trials, and peer-reviewed articles published in English that directly addressed antimicrobial regimens or surgical interventions in managing *Mycobacterium ulcerans* infections. Articles were excluded if they were not in English, lacked full-text access, or did not mention BU treatment strategies. Data extraction and synthesis emphasized identifying key themes and trends to provide a comprehensive and clinically meaningful narrative of current evidence and evolving practices.

## 3. Results and Discussion

### 3.1. Antimicrobial Management

#### 3.1.1. Current WHO-Recommended Regimens

The current antimicrobial management of *Mycobacterium ulcerans* infection is guided by WHO recommendations and reinforced by recent consensus guidelines from Australia, where BU incidence has notably increased in endemic regions such as Victoria and Queensland [3,10,23]. The standard treatment regimen consists of an eight-week course of rifampicin, combined with clarithromycin [10,23]. This dual oral antibiotic therapy has largely replaced injectable regimens involving streptomycin or amikacin, which are no longer recommended due to potential renal and ototoxicity [24,25,26]. Phillips et al. conducted an open-label randomized study comparing fully oral therapy with standard streptomycin-rifampicin, showing non-inferiority of rifampicin/clarithromycin over rifampicin/streptomycin in healing rates at 52 weeks (95.4%, IQR: 90.7–98.1% vs. 95.9%, IQR: 91.3–98.5%) [27]. The study found that the median time to healing was 24 weeks (IQR: 8–28) for streptomycin/rifampicin and 16 weeks (IQR: 8–25) for clarithromycin/rifampicin, with significantly more ototoxicity in the streptomycin group, suggesting streptomycin should no longer be recommended for *Mycobacterium ulcerans* infection treatment [27]. According to WHO recommendations, rifampicin at 10 mg/kg daily (up to 600 mg) plus clarithromycin 7.5 mg/kg twice daily (up to 500 mg twice daily) is the preferred combination, balancing efficacy and tolerability [3,11].

In Australia, a combination of rifampicin with fluoroquinolones, such as moxifloxacin and ciprofloxacin, is routinely used as a first-line treatment, although this regimen has not yet been evaluated in randomized controlled trials [11]. Fluoroquinolones exhibit strong bactericidal activity by disrupting bacterial DNA replication, with consistent efficacy demonstrated across laboratory and animal models [28,29,30,31]. Clinical data from Australia and other regions confirm their role in achieving lesion resolution, often without the need for extensive surgical intervention [31].

Fluoroquinolones are generally avoided in children and pregnant women due to concerns about cartilage toxicity as observed in animal studies [32,33,34]. Other adverse effects, such as tendinopathy, QTc prolongation, and neuropsychiatric symptoms, can occur across all age groups and are not unique to these populations, and notably, clarithromycin carries similar risks and, likewise, warrants careful monitoring [3,32,33,34,35,36,37]. However, as suggested by Muhi et al., a routine electrocardiogram is recommended, especially for patients at increased risk of arrhythmias [3]. In practice, fluoroquinolones have demonstrated comparable efficacy and tolerability to clarithromycin in both observational studies and clinical experience and remain a strong therapeutic alternative with a favorable benefit–risk profile when used in appropriate patient populations.

Macrolides, particularly clarithromycin, play a central role in BU treatment as part of the WHO-recommended oral regimens, which combine rifampicin with clarithromycin for eight weeks (RC8). This regimen has demonstrated non-inferior efficacy to the previous injectable streptomycin–rifampicin regimen in healing early lesions [3]. Macrolides bind to the 50S subunit of the bacterial ribosome, inhibiting protein synthesis and thereby preventing bacterial growth [38,39,40,41,42,43]. Its minimum inhibitory concentration (MIC) against *Mycobacterium ulcerans* is around 0.12 μg/mL [40,41,42,43]. Clarithromycin has demonstrated effective activity against *Mycobacterium ulcerans*, both in vitro and in vivo, showing synergistic bactericidal effects when combined with rifampicin. Clinical trials have reported cure rates exceeding 90% [3,44,45,46,47]. Clarithromycin and rifampicin act synergistically against *Mycobacterium ulcerans* by targeting distinct bacterial processes—protein synthesis and RNA transcription—leading to enhanced bactericidal activity, especially in slow-growing bacilli [44,45,46,47]. Their combined use also improves tissue penetration and reduces the risk of resistance, contributing to high cure rates observed in clinical trials [44,45,46,47].

However, drawbacks include twice-daily dosing (7.5 mg/kg per dose), which complicates adherence compared to once-daily alternatives, such as moxifloxacin, and drug interactions due to clarithromycin’s CYP3A4 inhibition, potentially exacerbating toxicity when combined with other metabolized medications, such as statins or anticoagulants [3,48,49]. While the RC8 regimen minimizes injection-related risks such as streptomycin-induced ototoxicity [38], clarithromycin carries a risk of gastrointestinal side effects and, more rarely, hepatotoxicity, warranting routine monitoring of liver enzymes during treatment [50,51,52]. Although large population-based studies by Björnsson et al. and Chalasani et al. did not identify clarithromycin as a major contributor to drug-induced liver injury [53,54], Australian cohort data reported hepatitis in 17% of severe antibiotic complications, indicating that clinically significant hepatotoxicity may be more common in this setting [37].

**Figure 1:**
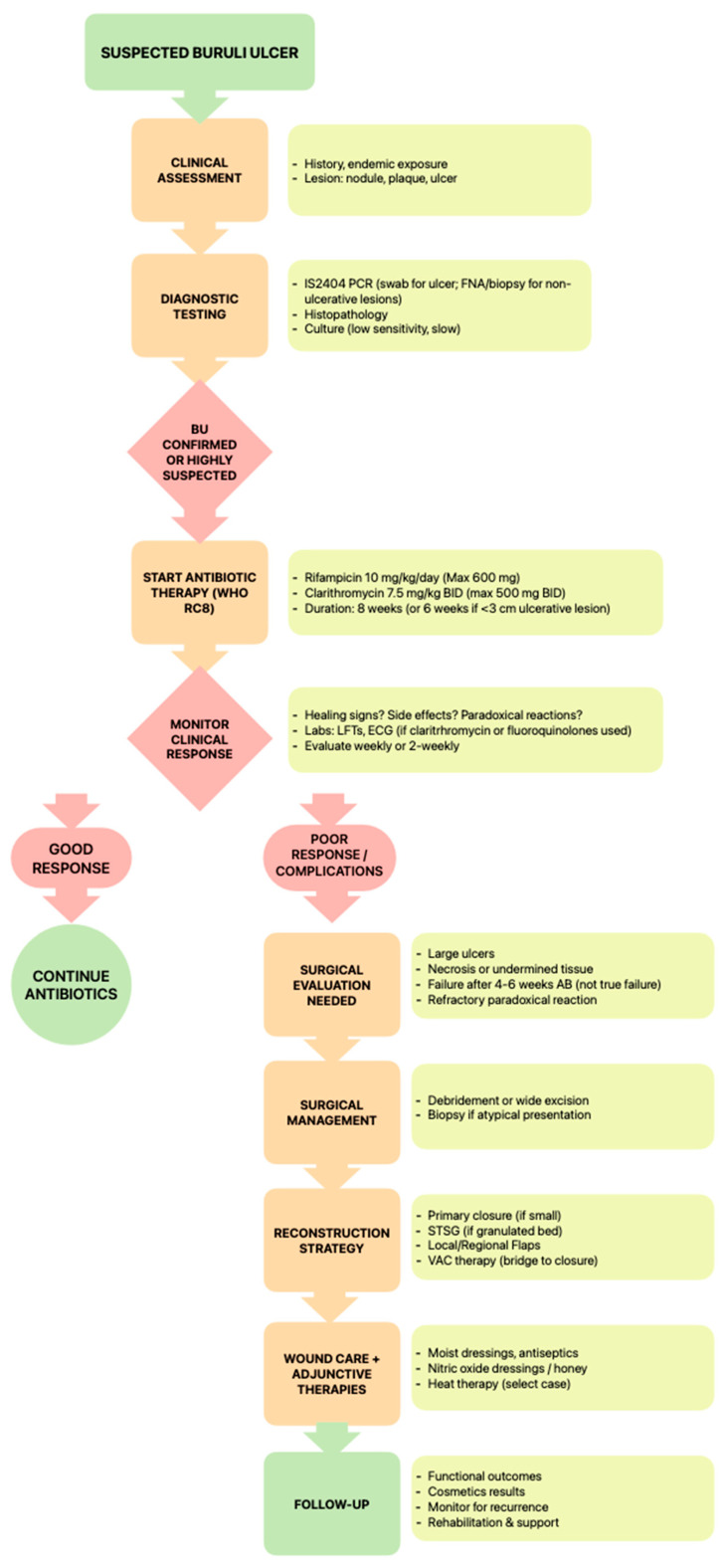
A proposed algorithm outlining the management approach for *Mycobacterium ulcerans* infection.

Treatment failure risks are heightened in immunocompromised patients, males, and those weighing > 90 kg, possibly due to suboptimal drug penetration, metabolic variability, or reduced clarithromycin levels caused by rifampicin-induced hepatic enzyme activity [3,55]. O’Brien et al.’s prospective study from Barwon Health identified male sex, weight > 90 kg, and immunosuppression as significant predictors of antibiotic treatment failure for *Mycobacterium ulcerans* (*p* < 0.001, 0.02, and 0.04, respectively), potentially due to lower per-kilogram dosing and highlighting the need for further pharmacokinetic research [55]. Despite these limitations, clarithromycin remains the preferred option over azithromycin due to more substantial evidence, although emerging drugs such as telacebec may eventually supplant macrolides in next-generation regimens [39]. A proposed algorithm outlining the management approach for *Mycobacterium ulcerans* infection is presented in Figure 1.

#### 3.1.2. Drug Resistance Concerns, Duration, and Compliance Challenges

While drug resistance remains theoretically possible, no cases have been reported in Australia, and confirmed resistance globally is rare [3]. The use of combination therapy mitigates the risk of resistance emergence, and monotherapy is strongly discouraged. Treatment duration is generally eight weeks but may be shortened to six weeks in select immunocompetent patients with small ulcerative lesions (<3 cm diameter), especially if combined with surgical debridement [3,55]. However, longer or tailored courses may be necessary for immunocompromised individuals, those with larger lesions, or those requiring steroid treatment for paradoxical reactions [3]. Compliance challenges can arise due to the prolonged treatment course, potential side effects, and significant drug–drug interactions. Many BU antibiotics, including rifampicin and clarithromycin, interact with commonly prescribed medications, which can complicate management, particularly in older patients with comorbidities, as often seen in the Australian setting. Adverse effects such as gastrointestinal intolerance and hepatotoxicity may also persist for weeks or months after antibiotic completion [3,50,51,52]. Close clinical monitoring, medication review, patient education, and support are essential to optimize adherence and outcomes.

#### 3.1.3. Role of Topical Versus Systemic Agents

The role of topical agents in BU management is adjunctive, rather than primary. Topical wound care, including cleaning, moist dressings, and sometimes honey or antiseptics, supports healing and prevents secondary infection; however, it does not address the underlying mycobacterial infection [3]. Nitric oxide-releasing dressings are a newer adjunct that shows promise for accelerating wound healing and reducing bacterial load due to NO’s antimicrobial and tissue-repair properties [3]. Phillips et al. conducted the first controlled trial, showing that topical creams releasing nitrogen oxides significantly accelerate the healing of BU lesions compared to a placebo, with minimal side effects, primarily reversible skin discoloration, suggesting a safe and effective non-surgical treatment option for rural settings [56]. Adjei et al. reported successful treatment of BUs in three pediatric patients using phenytoin powder; however, the small sample size limits the conclusiveness of the findings [57].

Localized heat therapy, which exploits the temperature sensitivity of *Mycobacterium ulcerans*, has also shown substantial benefit. Early studies confirmed its ability to inhibit bacterial growth in vitro and demonstrated complete lesion healing in small clinical cohorts without the need for surgery [58,59]. More recently, a Phase II trial in West Africa reported cure rates exceeding 80% in 53 patients treated with local heat alone, without adjunctive antimicrobials or surgical intervention [60]. Despite its promise, routine use in tropical settings remains limited by logistical challenges, the need for temperature-controlled equipment, and patient tolerability issues.

#### 3.1.4. Emerging Therapies

Cotrimoxazole has been evaluated as a potential treatment for BU, but evidence for its efficacy is limited. Yotsu et al. reviewed drug treatments for BU that assessed sulfamethoxazole/trimethoprim [61]. However, they only found one RCT by Fehr et al. involving 12 participants, in which cotrimoxazole reduced ulcer size by an average of 10.9% compared to a 24.5% increase with placebo (*p* = 0.15), with greater granulation tissue coverage observed in the treatment group (92% vs. 57%, *p* = 0.17) despite larger baseline ulcers [61,62]. Ultimately, Yotsu et al. concluded that cotrimoxazole did not treat BU [61]. While cotrimoxazole has demonstrated in vitro activity against *Mycobacterium ulcerans*, evidence supporting its effectiveness remains limited and methodologically weak [24,63]. A pilot study conducted by Espey et al. found that patients treated with dapsone and rifampicin had a greater reduction in BU size than the placebo group [64]. However, the initial ulcers were larger in the treatment group, and the difference in overall improvement was not statistically significant [64]. Their role in BU treatment is currently inconclusive and warrants further investigation.

Clofazimine, traditionally used for leprosy and increasingly explored for tuberculosis, has also been evaluated for BU. In a murine model, Converse et al. found that the combination of rifampicin plus clofazimine (RIF+CFZ) was effective against *Mycobacterium ulcerans* [65]. While RIF+CFZ initially acted more slowly than rifampicin–streptomycin (RIF+STR) or rifampicin–clarithromycin (RIF+CLR), it ultimately achieved comparable cure rates to RIF+STR and was superior to RIF+CLR in preventing relapse. Notably, none of the mice treated with RIF+CFZ for six or eight weeks had detectable bacteria, and relapse rates were low (5% for RIF+CFZ vs. >50% for RIF+CLR) [65]. However, clofazimine monotherapy was ineffective [65]. Similarly, a double-blinded RCT by Revill et al. found that clofazimine alone in surgically treated patients did not show benefit in shortening disease course or reducing surgical interventions, indicating that clofazimine should not be used as monotherapy [66].

In preclinical studies, telacebec (Q203), a cytochrome bc1:aa3 oxidase inhibitor, has demonstrated extraordinary potency against *Mycobacterium ulcerans* [67,68]. In vitro, Q203 has extremely low minimum inhibitory concentrations, and in mouse footpad models, Q203-containing regimens (often combined with rifapentine, clofazimine, or bedaquiline) rendered nearly all footpads culture negative after just two weeks of treatment [67]. No relapses were observed after this short course, compared to a 15% relapse rate with standard RIF+STR for four weeks. Q203 alone, or in combination with other agents, produced rapid and durable cures, with pharmacokinetic data indicating that plasma concentrations achievable in humans are sufficient for efficacy [67,68]. These results suggest that Q203 could enable ultrashort, all-oral regimens for BU, potentially reducing treatment duration from eight weeks to as little as one or two weeks [67,68]. Interestingly, a Phase 2 clinical trial of telacebec monotherapy in BU patients is currently underway in Australia, with preliminary results from 40 patients recently presented at the Second WHO Global Meeting on Skin Neglected Tropical Diseases in March 2025.

TB47, a novel diarylquinoline compound, has shown high bactericidal activity against *Mycobacterium ulcerans* in mouse models [69]. When administered at 0.8 mg/kg, Liu et al. found that TB47 alone was more effective than the current WHO-recommended regimen (rifampin plus streptomycin) [69]. TB47-containing oral regimens cured BU in as little as two weeks with daily or three weeks with intermittent dosing, preventing relapse in treated mice [69]. These findings highlight TB47’s potential as a core component of future, shorter, and fully oral BU treatment regimens.

While bedaquiline shows potential as a therapeutic option for *Mycobacterium ulcerans*, its high cost may limit widespread adoption, particularly in resource-constrained settings where BUs are most prevalent. Linezolid, although effective against various resistant mycobacteria, is generally deemed unsuitable due to its well-documented toxicity profile, particularly hematologic, neurologic, and metabolic effects such as lactic acidosis, which outweigh its use in non-lethal, chronic skin disease [70,71].

Beta-lactams are not currently a mainstay of BU therapy, but pharmacologic reviews discuss their use in combination regimens for *Mycobacterium ulcerans*. An in vitro study by Arenaz-Callao et al. revealed that while rifampicin showed no synergistic effects with existing anti-BU drugs, it demonstrated strong synergy with beta-lactam antibiotics, which also showed synergism with clarithromycin, another first-line BU treatment [72]. These results align with D’Agate’s findings, indicating a lack of interaction between rifampicin and clarithromycin in both in vitro and murine models [73]. The latter’s pharmacokinetic modelling and probability-of-target attainment analyses further supported the inclusion of amoxicillin/clavulanate in treatment regimens, identifying standard doses (22.5 mg/kg/1000 mg twice daily) as promising candidates for future efficacy trials [73]. A randomized controlled trial is currently underway in Africa to evaluate whether a four-week regimen of rifampicin, clarithromycin, and amoxicillin/clavulanate is non-inferior to the standard eight-week rifampicin–clarithromycin regimen [73]. Additionally, the data suggest that higher rifampicin doses may enhance treatment outcomes [73].

Current research on hyperbaric oxygen therapy (HBOT) for BU is limited but intriguing. Laboratory studies and a case report by Pszolla et al. have shown that *Mycobacterium ulcerans* prefers low-oxygen environments for optimal growth, as demonstrated by enhanced bacterial proliferation at reduced oxygen tension in culture systems [74,75]. This finding suggests that increasing tissue oxygenation may inhibit the growth of *Mycobacterium ulcerans*. Early animal studies support this hypothesis; Krieg et al. reported that hyperbaric oxygen treatment, combined with rifampicin and heat, improved outcomes in a murine model of *Mycobacterium ulcerans* infection [76]. However, clinical data remain sparse, and hyperbaric oxygen is not currently a standard therapy for BU. Most contemporary reports, including those from Japan, focus on antimicrobial regimens and do not mention HBOT as a routine or recommended intervention [77]. Thus, while HBOT has a plausible mechanistic rationale and some supportive preclinical data, further clinical research is needed to define its role in BU management [77].

In summary, standard WHO-endorsed treatments such as rifampicin–clarithromycin (RC8) are supported by high-level evidence and widespread use, while alternatives such as rifampicin–fluoroquinolone combinations are commonly used in practice, despite lacking RCT validation. Emerging drugs such as telacebec (Q203) and TB47 demonstrate promising efficacy in preclinical models and could substantially reduce treatment duration. The table also underscores important safety considerations, particularly for streptomycin, which is now discouraged due to ototoxicity. By synthesizing indications, efficacy, tolerability, and research maturity, this table serves as a concise clinical and research guide for optimizing BU therapy. Table 1 presents a structured comparison of antimicrobial regimens for Buruli ulcer, highlighting both established and novel treatments, serving as a concise clinical and research guide for optimizing BU therapy.

## 4. Diagnostic Challenges

The complexity of diagnosing *Mycobacterium ulcerans* infection is further compounded by the heterogeneous clinical manifestations and the variable sensitivity of diagnostic tests across different stages of the disease. Early lesions, such as nodules, plaques, or edema, often lack the classic ulcerative features, making clinical diagnosis challenging, even in endemic regions [5,78,79,80]. In these early stages, the lesion may not yet have ulcerated, and the bacteria are typically located in the subcutaneous tissue. As a result, superficial swabs may yield false-negative PCR results despite high bacterial burden, particularly in edematous lesions [81]. Consequently, clinicians must maintain a high index of suspicion and consider repeat testing or biopsy if initial results are inconclusive. This is particularly important because early diagnosis and timely initiation of therapy can reduce the extent of tissue destruction, enable shorter courses of antibiotics, and (in selected cases) allow for primary surgical excision with direct closure without the need for antimicrobial therapy [3].

In non-endemic regions, the rarity of the disease contributes to diagnostic delays, as healthcare providers may not be familiar with the clinical presentation of BU or the appropriate diagnostic algorithms [82]. Patients often undergo multiple consultations and treatments for other suspected conditions, such as bacterial cellulitis or fungal infections, before being considered for BU [83]. This delay worsens patient outcomes, increases healthcare costs, and complicates management. Education and awareness campaigns targeted at both clinicians and the community are essential to improve early recognition, encourage timely presentation to healthcare services, and facilitate prompt referral for specialized diagnostic testing.

Advanced molecular techniques such as real-time PCR have revolutionized the diagnosis of *Mycobacterium ulcerans* by providing rapid and specific detection of mycobacterial DNA [84,85]. These methods allow for earlier diagnosis compared to traditional culture, which requires up to 12 weeks of incubation due to the organism’s slow growth. Current guidelines recommend using swabs for ulcerated lesions and fine-needle aspiration (FNA) or punch biopsy for non-ulcerated lesions, depending on the clinical presentation [3]. Phillips et al. found that PCR targeting the IS2404 sequence on FNA samples had an 86% sensitivity for diagnosing *Mycobacterium ulcerans*, compared to 98% with punch biopsies. In comparison, culture and microscopy were significantly less sensitive (44% and 26%, respectively), supporting FNA-PCR as a viable, less invasive diagnostic method [84]. However, PCR requires well-equipped laboratories and trained personnel, which may not be readily available in resource-limited endemic settings [86,87]. To address this, point-of-care molecular diagnostics and simplified sample collection methods are under development, aiming to decentralize testing and facilitate earlier diagnosis in rural areas [88,89,90]. Histopathological examination remains a valuable adjunct, especially when molecular tests are unavailable or inconclusive [79]. Characteristic features, such as extensive coagulative necrosis with a minimal inflammatory response and the presence of acid-fast bacilli, can confirm the diagnosis [79].

## 5. Surgical Management

### 5.1. Debridement and Excision Margins

While introducing effective antibiotic regimens has shifted the primary management of BU away from surgery, operative intervention remains essential in selected cases [91]. These include patients with large ulcerative lesions, deep necrosis, extensive undermining, or failure to tolerate or respond to an adequate course of antibiotics [3,91,92]. Surgical debridement removes necrotic or undermined tissue that harbors residual mycobacteria and impedes healing [3,93]. This can help reduce bacterial load, hasten wound granulation, shorten recovery time, and reduce antibiotic duration [3]. For some patients, wide excision followed by primary closure, split-thickness skin grafting (STSG), or flap coverage may be necessary, especially when lesions exceed a critical size or when the skin cannot re-epithelialize on its own [92,93]. When patients cannot or prefer not to undergo the recommended oral antibiotic regimen, wide margin excisions for smaller lesions that can be closed via direct wound closure can be offered [3].

Furthermore, surgery may clarify the diagnosis in atypical or non-resolving cases, particularly where co-infection or malignancy is suspected. In paradoxical reactions (worsening of lesions despite appropriate antibiotics), excision can alleviate symptoms, improve wound healing rates, and prevent complications [91]. However, the timing of surgery relative to antibiotic initiation is still debated, and overt reliance on surgery can lead to unnecessary morbidity, highlighting the need for tailored, multidisciplinary decision-making [92,93].

### 5.2. Flap and Graft Option

The choice of reconstructive technique in BU management is influenced by the size, depth, and anatomical location of the defect, as well as the phase of treatment [94]. Larger ulcers, especially those resulting from extensive surgical excision or delayed presentation, often necessitate reconstruction using split-thickness skin grafts or local tissue flaps [95,96]. Skin grafting, when performed on a well-vascularized wound bed after adequate debridement and completion or concurrent antibiotic therapy, has shown favorable outcomes in endemic settings, with high graft take rates and reliable wound healing within a few weeks [95,96]. Local or regional flaps may be required to provide durable, vascularized coverage when the wound bed is compromised, such as with exposed tendon, bone, or areas of persistent inflammation [97,98].

Adjunctive modalities such as negative pressure wound therapy (NPWT) have also been employed to enhance granulation tissue formation, manage wound exudate, and optimize the wound environment before grafting or flap coverage. Murase et al. reported a case of BU in a woman in her 50s, who was successfully managed with a combination of antibiotics, surgical debridement, and NPWT, which facilitated granulation and allowed for successful skin grafting, ultimately avoiding amputation and achieving complete wound healing [99]. Loftus et al. presented a case report describing a non-healing BU that responded well to vacuum-assisted closure (VAC) dressings, resulting in complete healing without skin grafting. This further illustrates the potential of VAC therapy to enhance granulation tissue formation and manage wound exudate in BU care [100]. However, Kavanagh et al. highlight that while NPWT shows strong theoretical promise for controlling the necrotic and infected wounds characteristic of BU, its efficacy remains underexplored due to a lack of robust, quantitative studies, underscoring the need for randomized controlled trials to guide evidence-based practice [101].

### 5.3. Indications for Early Versus Delayed Reconstruction

The timing of surgical reconstruction in BU treatment requires careful consideration of infection status, wound evolution, and the patient’s overall condition. Early reconstruction may be feasible in cases of small, superficial ulcers where antibiotic therapy has rapidly controlled the infection and where debridement reveals healthy, well-vascularized tissue [3]. This approach can reduce hospital stay, expedite functional recovery, and minimize psychosocial impact, particularly in children and individuals with lesions in visible areas.

However, for more extensive or deep lesions, delayed reconstruction remains the preferred strategy [3,102]. Allowing sufficient time for the infection to resolve, the wound to demarcate, and necrotic tissue to declare itself helps avoid complications such as graft failure or recurrent ulceration. This staged approach also permits monitoring for paradoxical reactions, which can mimic disease progression and may necessitate further medical or surgical intervention [3].

Interdisciplinary coordination between surgical, infectious diseases, and wound care teams is crucial to optimize timing. Reconstruction should ideally be undertaken only when clinical signs of infection have resolved, antibiotic therapy is well underway (typically for at least 4–8 weeks), and the wound bed is suitably prepared [3,5,102]. Tailoring the timing to each patient’s presentation is essential to achieving durable wound closure and preserving function, particularly when lesions involve joints, facial structures, or weight-bearing surfaces.

**Figure 2:**
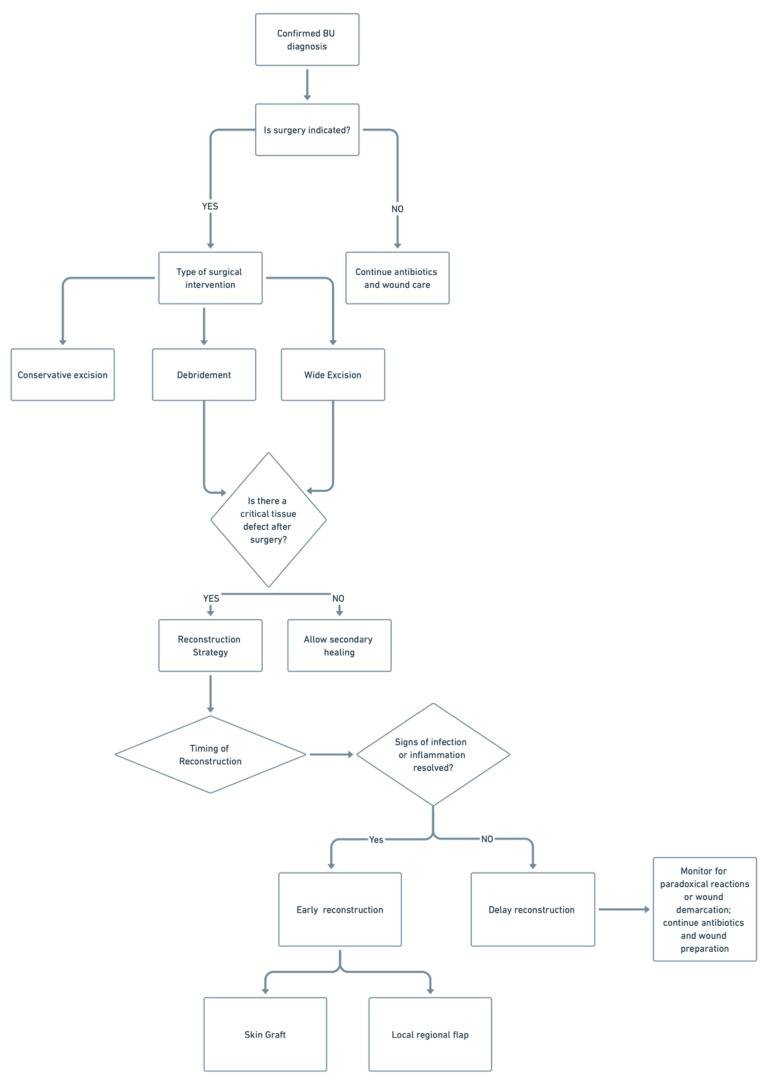
This figure outlines the surgical decision-making pathway for Buruli ulcer.

Surgical management of Buruli ulcer remains essential in selected cases, with procedures such as debridement, wide excision, and reconstruction guided by lesion severity, antibiotic response, and wound characteristics. Optimal outcomes depend on timing, appropriate use of grafts or flaps, and multidisciplinary coordination to ensure infection control and functional restoration. A summary flow chart of the role and types of surgical interventions is demonstrated in Figure 2.

## 6. Reconstructive Outcomes

### 6.1. Functional and Cosmetic Outcomes

Post-treatment functional recovery in BU is highly contingent on defect size, time to intervention, and the reconstruction method [103]. Small to moderate defects closed by primary suturing or split-thickness grafting generally restore near-normal range of motion in most cases, with patients regaining weight-bearing or fine-motor activities within 4–6 weeks [95]. In one long-term follow-up study of patients with limited BU lesions, 85% reported no lasting functional limitations in daily life [25]. Local and regional flaps, particularly fasciocutaneous designs, help maintain joint mobility and resist contracture, hastening the recovery process and facilitating a prompt return to activity [104]. Even extensive defects managed with free tissue transfer can achieve functional milestones (walking, grasping) in some patients when combined with early physiotherapy [97]. For instance, the use of ilioinguinal (regional) flaps in children achieved full weight-bearing by ~12 weeks post-op in all cases [97]. Overall, most BU patients reach near-normal movement and function after reconstruction.

Cosmetic outcomes correlate with reconstructive modality and anatomical site. Primary closures leave fine linear scars with an excellent color match. Split-thickness grafts often heal with hypopigmentation or textural irregularities, but careful donor-site selection and moisture, and silicone-based scar management limit aesthetic sequelae [3,105]. Graft cosmesis can be further improved with NPWT use in BU, preventing edema and contamination and thus the risk of complications [101]. Local flaps provide superior contour and color continuity, especially in facial or joint regions, though they introduce additional scar lines that can be concealed within natural skin creases [106]. Regional and free flaps carry a higher risk of color mismatch and bulkiness, occasionally necessitating staged debulking. Across all techniques, patient-reported satisfaction remains high when reconstruction is integrated with scar-mitigation therapies and counselling [107]. This underscores the value of combining surgical expertise with postoperative care.

Regardless of the method, early surgical intervention combined with antibiotics is crucial for optimal outcomes [95]. Delayed wound closure leads to larger scars and worse function [2,92]. In practice, a tailored approach should be used; smaller lesions may heal with simple excision or limited grafting, whereas complex cases are referred for further, more nuanced reconstructive options.

### 6.2. Limb Preservation Versus Amputation in Advanced Cases

In advanced BUs, defined by extensive soft-tissue necrosis, osteomyelitis, or large Category III ulcers, limb preservation remains the goal but is not always feasible [97,108]. Early, intensive rifampicin-based therapy combined with wide debridement and targeted reconstructive techniques (e.g., bone grafting, regional or free flaps) can salvage most limbs [95]. A series from Ghana reports salvage rates exceeding 90% when these measures are applied promptly [95]. However, when osteomyelitis is refractory, joints are irreversibly destroyed, or reconstruction repeatedly fails, amputation becomes the definitive intervention. Yet, even in these cases, antibiotic treatment and surgical debridement can facilitate conservative amputations, such as a below-knee amputation [100]. This can be followed by rehabilitation and prosthetic usage that restore mobility and function. For example, a Victorian case of BU osteomyelitis requiring below-knee amputation with timely rehab resulted in good recovery and excellent patient satisfaction [100].

### 6.3. Role of Multidisciplinary Care

Effective BU reconstruction hinges on a truly multidisciplinary, patient-centered approach. Infectious disease physicians initiate and monitor the antibiotic regimen, managing paradoxical reactions to optimize surgical success [3,35,36,37]. Plastic surgeons perform targeted excisions and design individualized reconstruction plans, selecting the appropriate graft or flap [3]. Wound care specialists oversee daily dressing changes, implement NPWT, and detect early signs of healing or complications. This can be optimized by having specialized nurses trained in BU wound care. Physiotherapists and occupational therapists can advise and initiate guided exercises at the bedside to prevent the risk of contractures and restore mobility [3,109,110]. Their role is particularly critical in the rare cases requiring amputation (Loftus et al., 2018 [100]). Finally, BU is a physical ailment and a psychosocial burden. Therefore, engagement with psychologists is essential to assist with the prolonged treatment, hospitalization, stigma, and disfigurement [111]. Programs in Australia and leading West African centers demonstrate that this integrated model achieves high cure rates, preserves function, and enhances patient satisfaction, underscoring the need for coordinated care to restore form fully and both physical and mental function.

## 7. Pediatric Considerations

### Special Approaches in Children (Common in Endemic Areas)

**Figure 3:**
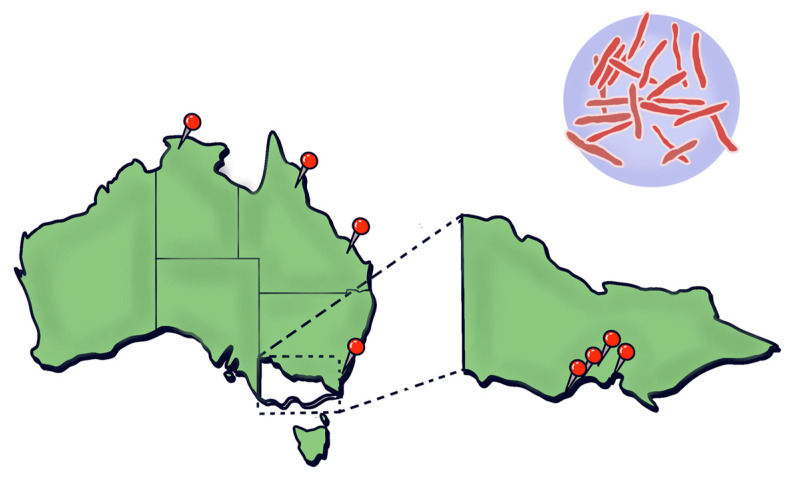
Sites of reported cases of *Mycobacterium ulcerans* in Australia.

BU poses distinct challenges in pediatric populations, especially in endemic regions of West Africa, where children under 15 years account for approximately 48% of all cases [112]. This high burden is primarily due to environmental exposure, such as during play in contaminated water sources, skin contact with soil, and low awareness of early BU symptoms in caregivers [2]. In contrast, BU in non-endemic high-income settings, such as southeastern Australia (Figure 3), predominantly affects older adults [3].

Diagnosing BU in children is often delayed. Early lesions, including nodules, plaques, and localized edema, may resemble insect bites or bacterial cellulitis and typically lack the hallmark ulceration, reducing suspicion of BU [3,113]. Furthermore, lower bacterial loads and non-ulcerated lesions in early disease stages decrease the sensitivity of polymerase chain reaction (PCR) and culture methods [1,81]. Non-invasive diagnostic techniques such as swab-based PCR and fine-needle aspirates are valuable in children, enabling sample collection with minimal discomfort [2,3].

The WHO-endorsed oral regimen of rifampicin and clarithromycin remains the standard of care for children, with dosages carefully weight-adjusted [3]. This combination has demonstrated high healing rates and a more favorable safety profile than earlier streptomycin-based regimens [27]. While previously used in combination therapies, Streptomycin has been associated with long-term ototoxicity, particularly high-frequency hearing loss in adults and children [26]. Fluoroquinolones such as ciprofloxacin and sparfloxacin have shown strong in vitro activity against *Mycobacterium ulcerans* [30], but their use in children is generally avoided due to potential risks of cartilage damage [33].

Surgical intervention is warranted in pediatric BU cases with large, necrotic, or non-healing lesions. In children, particular care is taken to minimize donor site morbidity, preserve joint mobility, and avoid disruption to growth plates during wound reconstruction. Split-thickness skin grafts and local flaps are commonly employed, and debridement may be required in the case of necrotic tissue or paradoxical reactions [3]. Functional outcomes are critical, and early physiotherapy is essential to prevent contractures and maintain limb function [97]. Cologne et al. also found that children treated with regional flaps, such as ilioinguinal, achieve full weight-bearing by approximately 12 weeks.

The psychosocial impact of BU on children should not be underestimated. Prolonged hospitalization, visible and sometimes permanent disfigurement, and school absenteeism contribute significantly to stigma, social isolation, and emotional distress [112]. These effects can lead to anxiety, low self-esteem, and reduced quality of life for affected children and their caregivers [112]. Integrating psychological support, peer counselling, and family-centered rehabilitation into BU care pathways has been recommended to mitigate these outcomes and support mental wellbeing during treatment and recovery [112]. Such interventions are particularly important in pediatric populations, where early psychosocial support can help prevent long-term psychological sequelae.

Prevention strategies in endemic areas should include educating parents and community members on early recognition of symptoms, school-based screening programs, and environmental interventions to reduce contact with contaminated water bodies and vegetation. Community surveillance and public health messaging are especially important in settings where BU is endemic, such as certain regions in West Africa and specific areas in Australia, where pediatric cases may occur due to environmental reservoirs like possums [2].

In summary, the management of BU in children requires an integrated approach that addresses diagnostic limitations, age-appropriate pharmacologic regimens, pediatric-adapted surgical techniques, and holistic psychosocial care. Tailoring care to the specific needs of this vulnerable population is essential for improving clinical and functional outcomes in endemic regions.

## 8. Research Gaps and Future Directions

### 8.1. Need for Randomized Controlled Trials on Antibiotic Duration

The available literature on randomized controlled trials on antibiotic duration for BUs is sparse. The current first-line standard management for Bus, as recommended by WHO, is the combination of rifampicin with clarithromycin [11,93]. To date, there have been two RCTs on antibiotics’ efficacy and duration [114]. The paucity of RCTs on the optimal duration of antibiotics was similarly observed in a previous systematic review [115]. Phillips et al. conducted an RCT in Ghana on patients with early BU lesions to evaluate the efficacy of oral rifampicin 10 mg/kg plus intramuscular streptomycin 15 mg/kg once daily for eight weeks compared to fully oral rifampicin 10 mg/kg plus clarithromycin 15 mg/kg extended release once daily for eight weeks. This trial found similar rates of healing of BU between both groups [27], thereby suggesting that fully oral therapy could be beneficial for patients with limited lesions and limited access to healthcare. Most studies have been conducted on mouse models [116], which might not directly translate to humans. Most studies investigating optimal antibiotic duration have been observational [56,117,118], all of which found that antibiotics may be effective in less than the recommended duration of eight weeks. Accordingly, this reinforces the need for RCTs investigating the possibility of shortening the duration of eight weeks of oral antibiotics to improve reliability.

### 8.2. Better Guidelines for Surgery Timing

The timing of surgery in the treatment of BUs is uncertain. Surgery was the main treatment of choice for BU before the WHO introduced recommendations for eight weeks of oral rifampicin and streptomycin in 2004. Since then, surgery has been recognized as an adjunct treatment. Yet, there is a lack of consensus on when to operate. An RCT investigating the effect of delaying surgical intervention for 14 weeks on healing rates without surgery found that postponing the decision to operate resulted in shorter hospital stays and wound care duration [92]. It was found that antibiotics alone without surgery could treat large ulcers and did not result in slower healing or greater impaired functional capacity [92]. However, this is contradictory to an observational study, which reported that surgery still plays a critical role in treating BU, resulting in higher wound healing rates and reduced antibiotic toxicity [91]. While the consensus is that aggressive surgery is no longer recommended [3], surgery may still be required in certain circumstances. Further research is needed to better identify when to operate and which patient subgroups may benefit the most from surgery, particularly those with immunosuppression, comorbidities, or higher recurrence risk.

It is important to consider that the timing of surgery may also be largely affected by social factors such as costs and access to healthcare for people living in remote, rural areas. There is a need to take into account social factors when deciding on guidelines for the timing of surgery [97].

### 8.3. Vaccine Potential

Vaccines are likely the only means to achieve widespread BU prevention or eradication due to the difficulty of eliminating the environmental source of infection. However, historically, BU vaccine development has been mired with challenges, with none reaching advanced widespread use. Several vaccine strategies have been explored: BCG provided ~47% short-term protection that wanes within a year, and live-attenuated *Mycobacterium ulcerans* or *Mycobacterium marinum* strains delayed but did not prevent disease in mice [119]. DNA vaccines encoding Ag85a induced strong Th1 responses and ~100-fold lower bacterial loads, yet remained less protective than BCG [119]. Protein subunit vaccines targeting proteins such as MUL_2232 and MUL_3720 generated high antibody titers without preventing ulceration, while vaccines targeting mycolactone synthase domains elicited IFN-γ/IL-2 responses but failed to lower bacterial counts [119].

However, BU vaccine development is now entering an optimistic phase, with new concepts that are effective in the laboratory. Researchers are focusing on multivalent vaccines that address the dual targets of bacteria and toxin [120]. The success of the Burulivac formulation in mice was highlighted by achieving sterile protection against *Mycobacterium ulcerans* infection [120]. In addition to human-targeted approaches, recent proposals have suggested that oral BCG vaccination of ringtail possums could reduce zoonotic transmission in endemic Australian regions, offering a novel approach to limiting environmental spread [121].

### 8.4. Public Health Interventions

Health education and community engagement are vital to the success of BU control programs. A robust understanding of the perceptions towards BU may improve health responses to reduce the disease burden amongst vulnerable populations in endemic regions. In many affected communities, lack of awareness and local misconceptions, for example, viewing BU as a curse or witchcraft, lead patients to seek traditional healers and delay proper treatment [122]. A study conducted in Ghana found misconceptions regarding the cause of the lesions, with causes thought to be witchcraft, curses, close contact, hygiene, and the environment [123]. Barriers to treatment were social stigma, financial hardship, and fear of treatment [123]. To counter this, WHO advocates information, education, and communication campaigns in endemic villages and schools, as well as training community health workers and school teachers to recognize it [124].

Engaging local leaders and former BU patients as advocates further builds trust in medical services. Notably, community-led support initiatives have improved outcomes: in one Ghanaian district, providing daily transport and meals to patients during outpatient antibiotic therapy dramatically increased early case detection and cut treatment default rates from over 50% to about 1.5% [125].

Furthermore, as early detection and active surveillance are critical to reducing disease morbidity, implementing such programs can significantly reduce the risk of delayed diagnosis. For example, an innovative surveillance method explored in Australia’s recent study showed that detecting *Mycobacterium ulcerans* in local possum feces can precede human cases by up to 39 months, providing an early warning of BU risk in the community [126].

Since the early 2000s, the mainstay of BU treatment has been a course of combination antibiotics, replacing the previous surgery-first approaches. This all-oral antibiotic protocol achieves cure rates exceeding 90% in patients who complete the full course [68]. Further, the switch to an all-oral antimicrobial treatment has significantly improved access to treatment and adherence to ongoing treatment, even for those in remote areas, with one study finding no reporting no association between distance to the clinic and treatment completion [127].

## 9. Conclusions

BU remains a significant global health challenge, particularly in resource-limited settings where early diagnosis and access to effective treatments are limited. Multidisciplinary approaches, combining timely antimicrobial therapy with appropriate surgical interventions, are crucial for achieving the best possible outcomes. Future research, including randomized controlled trials and investigations into emerging therapies such as telacebec, must refine management strategies and address gaps in current treatment protocols. By improving early detection, antibiotic treatment regimens, and reconstructive options, we can better mitigate the long-term functional and psychosocial impacts of this debilitating disease.

## Figures and Tables

**Table 1:** Summary of antimicrobial regimens for Buruli ulcer.

Antimicrobial Agent/Regimen	Indications	Efficacy	Side Effects	Evidence Level
Rifampicin + Clarithromycin (RC8)	First-line; WHO-recommended for all age groups	Cure rates > 90%; proven in RCTs	Gastrointestinal, hepatotoxicity, QTc prolongation, dysgeusia, and drug interactions	High (WHO and RCTs)
Rifampicin + Moxifloxacin	Alternative first-line in Australia (esp. adults)	Comparable to clarithromycin in practice	QTc prolongation, tendon issues (esp. elderly), photosensitivity	Moderate (lab + observational studies)
Rifampicin + Ciprofloxacin	Alternative option for children	Effective, supported by observational data	Similar to moxifloxacin, cartilage toxicity in children	Moderate (lab + observational studies)
Rifampicin + Streptomycin (Discontinued)	Previously first-line; now discontinued	Comparable efficacy, more side effects	High ototoxicity, nephrotoxicity	High (RCTs) but now outdated
Rifampicin + Clofazimine	Emerging; suitable in combination therapy	Effective in the murine model, relapse prevention	Skin discoloration, GI upset (rare)	Low (preclinical + murine models)
Telacebec (Q203)	Investigational; ultrashort regimen candidate	Sterile cures in mice; ongoing trials in humans	Minimal in small clinical studies	High (preclinical + Phase 2 ongoing)
TB47	Experimental; high activity in murine models	Cures BU in mice in 2–3 weeks	No severe reports in animals	Low (murine models)
Cotrimoxazole (TMP-SMX)	Investigational; weak evidence, small RCTs	No significant clinical benefit	Well tolerated, minor skin discoloration	Low (small RCT, observational)
Dapsone + Rifampicin	Pilot study, limited efficacy	Non-significant improvement over placebo	Can cause SJS, limited sample size	Low (pilot, not statistically significant)
Beta-lactam (Amox/Clavulanic Acid) + Rifampicin + Clarithromycin	Investigational; RCT underway	Potential four-week alternative: synergistic action	Minimal known so far	Moderate (in vitro + modeling, RCT ongoing)
Linezolid	Resistant TB; not favored for BU due to toxicity	Effective but high toxicity risk	Hematologic, neurologic, and lactic acidosis	Low (case reports, side-effect profile)
Bedaquiline	Investigational; high cost limits use	Promising in vitro/in vivo	Cost, QTc prolongation, liver impact	Low (preclinical, expensive)

## Data Availability

All data can be found within this manuscript.

## Abbreviations

The following abbreviations are used in this manuscript:

BU	Buruli Ulcer
NPWT	Negative Pressure Wound Therapy
FNA	Fine Needle Aspirate
PCR	Polymerase Chain Reaction
DNA	Deoxyribonucleic Acid
HBOT	Hyperbaric Oxygen Therapy
VAC	Vacuum Assisted Closure
RIF + STR	Rifampicin + Streptomycin
RIF + CLR	Rifampicin + Clarithromycin
RIF + CFZ	Rifampicin + Clofazimine
